# From Correlation to Causation: Understanding Episodic Memory Networks

**DOI:** 10.1007/s12264-025-01407-2

**Published:** 2025-06-24

**Authors:** Ahsan Khan, Jing Liu, Maité Crespo-García, Kai Yuan, Cheng-Peng Hu, Ziyin Ren, Chun-Hang Eden Ti, Desmond J. Oathes, Raymond Kai-Yu Tong

**Affiliations:** 1 Center for Brain Imaging and Stimulation, Department of Psychiatry, University of Pennsylvania, Philadelphia, PA USA; 2 Department of Applied and Social Sciences, Hong Kong Polytechnic University, Hong Kong, China; 3 MRC Cognition and Brain Sciences Unit, University of Cambridge, Cambridge, CB2 7EF United Kingdom; 4 Department of Biomedical Engineering, The Chinese University of Hong Kong, Hong Kong, China

**Keywords:** Transcranial magnetic stimulation (TMS), Episodic memory, Hippocampus, Medial temporal lobe, Brain stimulation, Functional magnetic resonance imaging (fMRI), Electroencephalography (EEG)

## Abstract

Episodic memory, our ability to recall past experiences, is supported by structures in the medial temporal lobe (MTL) particularly the hippocampus, and its interactions with fronto-parietal brain regions. Understanding how these brain regions coordinate to encode, consolidate, and retrieve episodic memories remains a fundamental question in cognitive neuroscience. Non-invasive brain stimulation (NIBS) methods, especially transcranial magnetic stimulation (TMS), have advanced episodic memory research beyond traditional lesion studies and neuroimaging by enabling causal investigations through targeted magnetic stimulation to specific brain regions. This review begins by delineating the evolving understanding of episodic memory from both psychological and neurobiological perspectives and discusses the brain networks supporting episodic memory processes. Then, we review studies that employed TMS to modulate episodic memory, with the aim of identifying potential cortical regions that could be used as stimulation sites to modulate episodic memory networks. We conclude with the implications and prospects of using NIBS to understand episodic memory mechanisms.

**Supplementary Information:**

The online version contains supplementary material available at 10.1007/s12264-025-01407-2.

## Introduction

Episodic memory enables us to mentally travel back in time and re-experience past events. Understanding the cognitive processes underlying episodic memory is not just a scientific puzzle concerning cognitive neuroscientists; this knowledge is also critical for developing intervention methods to treat patients with memory-related problems, such as those with Alzheimer’s disease, schizophrenia, post-traumatic stress disorder, and several other neurological and psychiatric disorders. Recent advancements in neuroscience have significantly enhanced our understanding of episodic memory processes and identified cortical regions within the frontal, parietal, and temporal lobes that form a crucial network for encoding, consolidating, and retrieving memories [1–5]. However, the precise functional roles of these brain regions and their interactions in episodic memory, as well as their causal contributions, remain a subject of ongoing research. This review explores the evolution of our understanding of episodic memory networks, examining both correlational and causal evidence to highlight progress and suggest future directions.

Much of our earlier understanding regarding the functional roles of different brain regions in episodic memory came from lesion studies. The famous Henry Molaison (H.M.) case is a perfect example: this patient unintendedly suffered from severe episodic memory loss after the removal of hippocampal structures, pointing to the hippocampus as the hub of episodic memory processes [6]. Similarly, other studies investigated how lesions in the frontal and parietal parts of the brain impair verbal or spatial aspects of episodic memory [7, 8], suggesting their potential supplementary role in episodic memory processes. Functional neuroimaging methods such as functional magnetic resonance imaging (fMRI), positron emission tomography (PET), Magnetoencephalography (MEG), and Electroencephalography (EEG) further expanded our understanding by providing correlational evidence of memory functions with brain regions [9, 10]. Lesion and neuroimaging studies were complemented by the use of non-invasive brain stimulation techniques in the late 20th century. These techniques, such as transcranial magnetic stimulation (TMS) and transcranial electrical stimulation (TES) [11, 12] allowed researchers to directly stimulate brain regions to understand their causal role in episodic memory and potentially enhance memory function. Among these, TMS garnered significant attention because of its precision in targeting specific brain regions, its safety profile, and its user-friendly nature. These attributes contributed to its Food and Drug Administration (FDA) approval for treating Major Depressive Disorder (MDD), Migraines with Aura, Anxiety comorbid with MDD, and Obsessive Compulsive Disorder (OCD) [13]. Given that the human brain functions as a network [14], stimulating a specific brain region not only impacts that region locally but also exerts network-wide effects, reaching distant brain areas [15]. Thus, TMS allows for network-wide stimulation, introducing the exciting prospect of modulating deeper brain structures like the hippocampus indirectly, which plays a critical role in episodic memory processes.

In this comprehensive review, we start with a historical outline of pivotal studies that shaped our understanding of episodic memory processes, while considering both psychological and neurobiological perspectives. We then examine key brain regions within the memory network, specifically focusing on how these regions contribute individually and as part of the network to episodic memory processes. Following this, we systematically search for studies targeting different nodes in the episodic memory network using TMS, with the goal of identifying potential cortical sites that could serve as promising targets for stimulation studies, following a systematic-narrative review approach. We finally discuss future directions of using brain stimulation for understanding and potentially improving memory function in patients with memory deficits. Overall, the article provides a comprehensive narrative that integrates historical, empirical, and translational aspects of episodic memory research.

## The Discovery of the Episodic Memory System

In the early 1950s, a patient suffering from epilepsy went through a surgical procedure in which the hippocampus and adjacent structures were removed to alleviate epileptic seizures. While the surgery was successful in reducing seizures, unintendingly, it also resulted in severe retrograde and anterograde amnesia [6]. This study provided conclusive evidence of the involvement of the hippocampus in long-term memory processes and later became famous as the H.M. study. However, until this point, episodic memory was not considered a separate memory system. It was only later in 1972 that Endel Tulving contrasted semantic memory to another system of memory which he called episodic memory, and proposed that semantic memory is primarily associated with understanding language and episodic memory for remembering events [16]. Tulving defined episodic memory as a system that receives and stores information about specific episodes or events, along with the temporal and spatial relationships among them. In his book “Elements of Memory”, he suggested that these two systems are functionally distinct [17]. This influential proposal by Tulving laid the groundwork for future research on episodic memory.

**Fig. 1:**
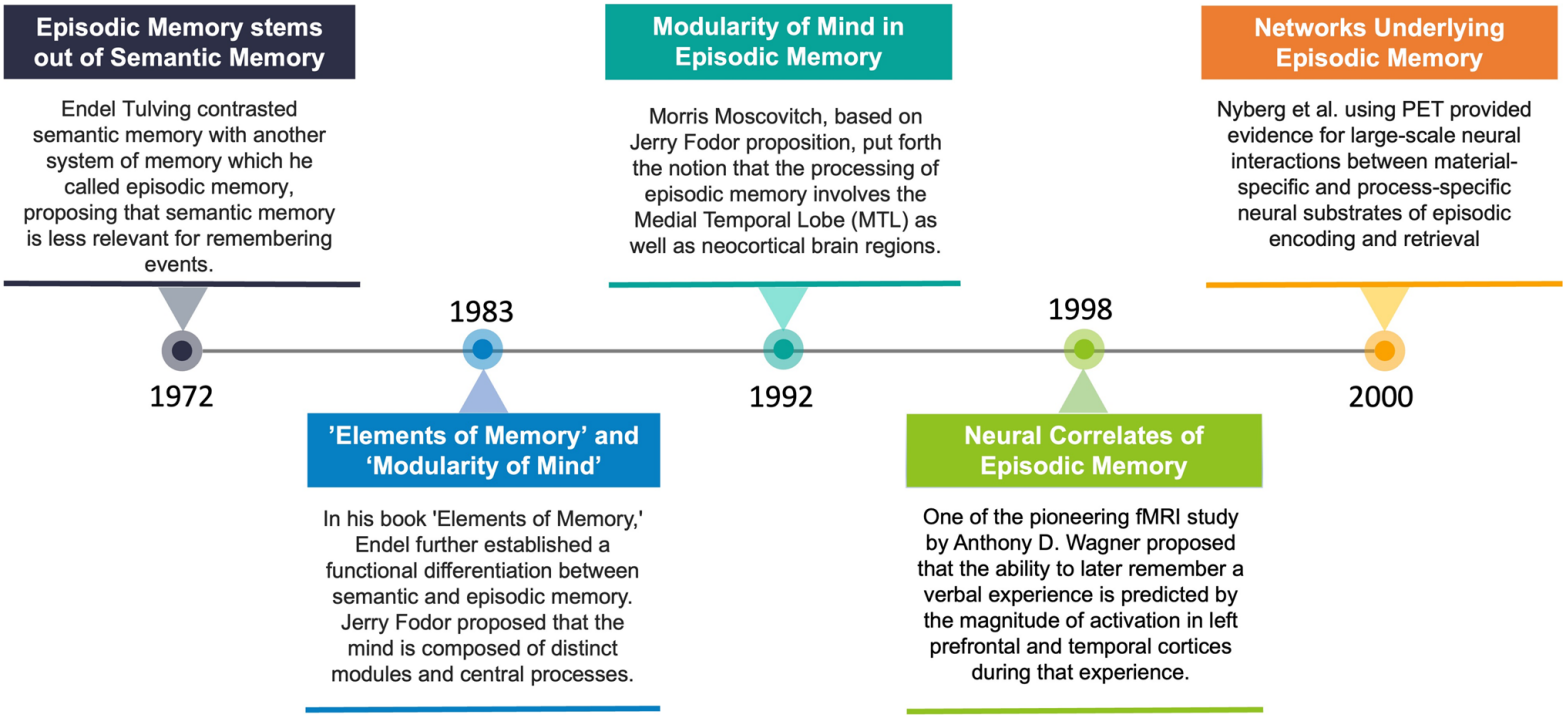
A brief overview of foundational studies in episodic memory research during the 20th century.

Around the same time, Jerry Fodor published "The Modularity of Mind," proposing that the mind is organized into distinct input systems that receive different inputs from the environment, which are then evaluated and responded to by central systems [18]. Building upon Fodor's theory, Morris Moscovitch proposed a neuropsychological model of memory in 1992 that incorporated modules and central systems [19]. According to this model, the processing of episodic memory occurs in groups of neurons distributed throughout the medial temporal lobe (MTL) and neocortex. One of the earliest studies investigating the neural markers of episodic memory encoding and retrieval was conducted in 1996 by Nyberg *et al*. [20]. The study utilizing PET pointed out general and specific brain areas recruited for processing specific aspects of remembered events, with left frontal regions more involved in encoding while right frontal regions in retrieval. The study further provided evidence for information-specific activations. For example, during encoding, stronger activation was observed for item information in the left hippocampus, for location in the right parietal, and for time in the left fusiform. During retrieval, activation was stronger for item information in the right inferior frontal and temporal regions, location in the left frontal, and time in anterior cingulate cortices. Wagner *et al.'s* fMRI study provided further evidence for this network model, suggesting that stronger activation in the left prefrontal and temporal cortices during encoding correlated with better memory retrieval [21]. A follow-up PET study by Nyberg *et al.* demonstrated that both material-specific regions (like those for remembering pictures or sentences) and process-specific regions (like those for encoding or retrieval) interact in large-scale networks to support episodic memory processes [22]. These earlier findings collectively highlighted that episodic memory is not confined to a single region but emerges from interactions among distributed brain regions. Fig. 1. provides a brief overview of foundational studies in episodic memory research during the 20th century.

## Role of Distinct Brain Regions in Episodic Memory Processes

While these milestone studies identified that frontal, parietal, and MTL regions play a key role in episodic memory function, the mechanistic understanding of the processes remained a central inquiry. Particularly, how these brain regions support different episodic memory processes including encoding, short-term maintenance, consolidation, and retrieval. In this passage, we briefly outline our current understanding of how different brain regions contribute to episodic memory processes.

### Episodic Memory Encoding

Episodic memory encoding is the process through which the brain first registers an episodic experience. During the encoding phase, episodic information, including sensory details and immersive gist, is first coded in the domain-specific sensory brain regions. This information binds together to form a labile initial memory representation or an engram within the hippocampus [23, 24]. Notably, effective encoding requires the coordination of several cognitive processes, including selective attention to the targeted items and using cognitive control to ignore the irrelevant distractors from the surrounding environment. These general cognitive processes are primarily supported by the prefrontal cortex (PFC) and the parietal cortex [25, 26]. Specifically, regions in the PFC such as the lateral prefrontal cortex (LPFC) and medial prefrontal cortex (mPFC) regions play a key role in the control processes that facilitate the encoding of episodic memories [27]. The PFC works in coordination with the hippocampus to integrate new information into existing memory structures [28, 29]. The role of attentional mechanisms mainly mediated by the parietal cortex and their interaction with hippocampal structure is also crucial for the formation and retrieval of episodic memories. For example, it has been reported that attention stabilizes hippocampal representations of memories that are correctly remembered [30, 31]. Thus, the encoding of episodic memories involves sensory brain regions, the hippocampus, and the PFC, with effective encoding requiring selective attention and cognitive control supported by the prefrontal and parietal cortices. For a detailed review of episodic memory encoding processes, please see [32]

In episodic memory research, a common observation is that memories associated with emotional content, be it positive or negative, tend to be recalled with greater vividness compared to neutral memories [33]. This may occur due to the arousing nature of the emotional stimuli that directs our attentional resources to the stimuli [34] and facilitates the encoding of information by allocating resources to the emotional content [35]. This enhanced recall of emotional memories is linked to the interplay between the hippocampus and the amygdala with a stronger connectivity observed between these two regions for emotional memories [36, 37]. While the amygdala is not traditionally considered part of the core episodic memory network, it plays a crucial role in processing emotional aspects of memories.

### Short-Term Maintenance

During the post-encoding short-term maintenance period which typically lasts only a few seconds, the newly formed episodic memory representations are reactivated via the bidirectional hippocampal-neocortex networks [38, 39]. The active maintenance or rehearsal of memory representations during this period is crucial for preventing the decay of the encoded information [40]. This active maintenance requires the internal direction of attention to sustain the memory traces and regions in the parietal cortex are involved in directing top-down attention towards the internal representations of the memory, effectively "refreshing" the memory traces [4]. It has been further demonstrated that neural representations during retrieval are more similar to the post-encoding short-term maintenance than to representations during encoding [38]. Thus, short-term maintenance plays a crucial role in how episodic memory representations are transformed and how they are later remembered.

Short-term maintenance shares similarities with working memory as they both involve maintaining information in the brain, however, working memory also involves actively manipulating that information to carry out the task at hand. One common mediator in both functions is the involvement of prefrontal regions. Particularly, the dorsolateral prefrontal cortex (DLPFC) is believed to contribute to long-term memory formation through its role in the organization of working memory [41]. For an extensive review of how the DLPFC contributes to working memory and episodic memory, see [42, 43]

### Episodic Memory Consolidation

Episodic memory consolidation refers to the process through which the newly formed memory traces are further stabilized and stored for later successful retrieval. This consolidation is broadly divided into two processes including synaptic consolidation and system consolidation [44]. Synaptic consolidation is thought to occur at a cellular level immediately after encoding and can last for hours, while system consolidation refers to the process in which these memories are reorganized and restructured in a way that they become less dependent on the hippocampus and more on cortical regions [44]. According to the widely accepted systems consolidation theory, the transformation of memories into non-hippocampal dependent traces is essential for them to be remembered. The model further suggests that the system consolidation occurs during offline periods including both awake rest and sleep, but predominantly during sleep [45]. During sleep, the newly formed memory representations are repeatedly reactivated via the precise coupling between the hippocampal ripple activity, thalamocortical spindles, and neocortical slow oscillations [45]. This repeated reactivation during non-rapid eye-movement (NREM) sleep supports the transformation of memories from the temporally labile, hippocampal-dependent formats into more decontextualized, stable formats that are long-term stored in the neocortex [46–48]. For a review of how memory consolidation differs between wakefulness and sleep, see [47].

An alternate account of episodic memory consolidation is based on context binding which suggests that the hippocampus binds the item information and the context information that make up the episodic event and that this consolidation does not happen in the cortex [49, 50]. The key distinction between context binding theory and system consolidation theory lies in their perspectives on the role of the hippocampus. Context binding theory suggests that the role of the hippocampus is not temporary; rather, it plays a continuous role in supporting context-dependent memories such as episodic memory, while the neocortex supports context-independent memory representations. Importantly, the context changes with time and thus the episodic experience extends in time and includes time periods before and after the item is presented, an important part that is missing in the memory consolidation account of episodic memory encoding. This temporal extension in time also impacts what will be eventually forgotten. Anything that occurs temporally close to the item being studied within the same context will later interfere with the target memory and can cause forgetting. Thus, the two accounts of episodic memory consolidation agree on the larger part that consolidation is a pre-requisite for a memory to be later remembered, however, they diverge on the roles of the hippocampus and cortical regions in the process. For a detailed review of context binding and its association with existing models see [49].

There has been a general consensus among the neuroscience community that the transfer of short-term memory to long-term memory takes place through the transfer of memory information from the hippocampus to cortical regions [33, 46–48]. However, the study by Kitamura *et al.* found that during the early stages of learning, neurons responsible for contextual fear memory rapidly activated in both the hippocampus and PFC [51]. Thus, memories of the event were being stored in both these regions at the time of encoding. The study further reported that prefrontal neurons strengthen their role in memory expression over time, while hippocampal neurons gradually lose this function. Overall, the study challenges the idea of the transfer of memories from MTL regions to cortical regions, instead, it suggests that memory traces are already present in the cortical regions along with the hippocampus and there is a shift in the balance that happens during consolidation processes. Similar findings have been reported by Brodt *et al.*, in which they found that new memory traces are rapidly encoded into the cortical regions particularly the parietal cortex, challenging traditional views of systems memory consolidation [52]. The process of memory consolidation, despite its critical role in episodic memory, remains insufficiently understood and requires further investigation.

### Episodic Memory Retrieval

At retrieval, effective internal or external cues result in the retrieval of memory representations stored in the neocortex [23, 53]. Earlier research studies advocated that episodic memory retrieval is dependent and to some extent functionally similar to episodic memory encoding [54]. One of the pioneering theories, namely transfer-appropriate processing (TAP) proposed by Morris *et al.* lays the foundation of episodic memories on two fundamental principles: firstly, memories are encoded based on the cognitive processes involved when an event is initially experienced, and secondly, effective memory recall takes place when these initial cognitive processes are re-enacted [55]. TAP is further supported by neurobiological models that show shared brain regions and similar neural activity patterns activated during encoding and retrieval of memories [56, 57]. Specifically, a retrieval cue triggers the memory traces stored in the hippocampus, further leading to the reinstatement of encoded memories [58, 59]. For a discussion on the interplay between encoding and retrieval processes, see [60]. In contrast, more recent neurobiological models propose that retrieval is not simply a reinstatement of the neural activity patterns from the encoding phase; instead, it is a constructive or reconstructive process [59, 61]. Retrieval cues trigger the hippocampus to reconstruct the consolidated neocortical elements into coherent events. This reconstruction process leads to transformed neural representations during memory retrieval via abstraction, integration, or forgetting. However, according to the hippocampal memory indexing theory, hippocampal representation is an index or code that contains information about the episodic experience distributed in the cortical and subcortical brain regions [62, 63]. During retrieval, a partial cue can activate this hippocampal index resulting in the activation of neocortical patterns associated with that specific index of the episode and thus retrieve the associated memory. For an in-depth review of memory indexing theory, refer to [50].

During encoding and short-term maintenance processes, brain regions responsible for general cognitive control are active during the retrieval process [4, 64]. For example, inhibitory control mechanisms mediated by PFC are thought to contribute to the successful retrieval of target memories while inhibiting non-target memories [65, 66]. In addition, the parietal cortex did not reach much attention in earlier accounts of episodic memory mainly because lesions in the parietal cortex do not cause severe episodic memory impairments [8, 67]. However, activation in the regions in the parietal cortex has been consistently observed in neuroimaging studies, particularly during retrieval of episodic memories [5]. These parietal regions also exhibit shared connections with regions in the PFC and the MTL, including the hippocampus. Notably, the human hippocampus demonstrates a robust functional connection with the ventral region of the parietal cortex. The Attention to Memory (AtoM) model, proposes a two-way interaction between the parietal cortex and hippocampus during memory retrieval [4]. According to AtoM, the parietal cortex directs top-down attention to retrieve memories. In turn, the retrieved memories themselves demand bottom-up attention, further strengthening the retrieval process. Instead of proposing a specific role for the parietal cortex in episodic memory, the model suggests it has a more general role in attention. This general attentional role can then be applied to episodic memory retrieval by directing focus through specific sub-regions within the parietal cortex itself.

**Fig. 2:**
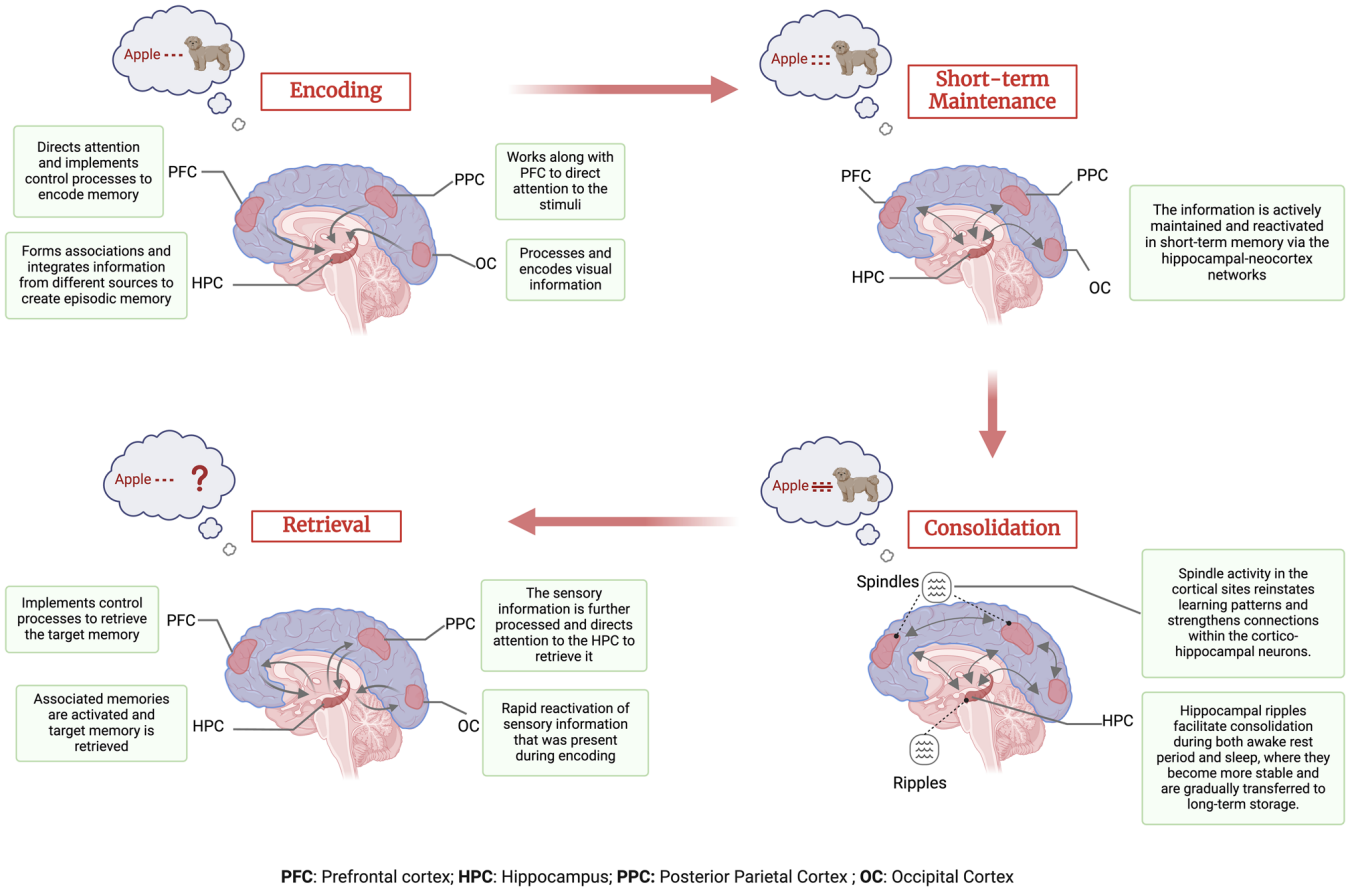
The Figure shows a simplified model of the episodic memory process from encoding to the retrieval stage. During the process of encoding, the presentation of a stimulus event (for example the word Apple shown with an image of a dog, and participants are instructed to remember the association) triggers the activation of a wide range of cortical regions in the frontal, parietal and occipital brain regions. These brain regions direct the information to the hippocampus to encode it. This episodic information is actively maintained in the short-term memory through a repeated interaction between hippocampal and neocortical brain regions which is then consolidated. During consolidation, sleep spindles in cortical regions act as a bridge between the cortex and hippocampus, triggering the activation of ripples in the hippocampus. These ripples then strengthen connections within the hippocampus and project back to the cortex, reinforcing the learned information in cortical networks. When a retrieval cue (for example the word Apple) is presented later on, it initiates a partial reinstatement of the initial cortical activity pattern. This reactivated pattern then propagates to the hippocampus. The overlap between the activity generated by the retrieval cue and the stored cortical activity pattern causes the hippocampal representation to be reactivated. This, in turn, leads to a complete reinstatement of the original cortical activity pattern. Separate bi-directional arrows represent these two separate phenomena. Created in BioRender. Khan, A. (2025) https://BioRender.com/g49l145.

The functioning of the episodic memory network is achieved through the coordinated actions of distinct brain regions which are shared during different stages of episodic memory*.* Fig. 2 provides a summary of these processes, encompassing encoding, short-term maintenance, consolidation, and retrieval.

## Wired for Remembering: Episodic Memory Networks

Although individual brain regions are vital to episodic memory processes, it is the coordinated activity across various parts of the brain, referred to here as episodic memory networks, that truly underpins these processes. Thus, episodic memory networks refer to the interconnected brain regions that are involved in encoding, short-term maintenance, consolidation, and retrieval of episodic memories. Researchers have used various neuroimaging methodologies, including fMRI, PET, MEG, and EEG, to understand how these regions contribute to episodic memory processes as a network. Over time, several conceptual frameworks have emerged, each attempting to elucidate the collaborative roles of these brain regions. In the following sections, we discuss these frameworks to develop a better understanding of the collaborative nature of episodic memory.

### The MTL Memory System

The late 20th century saw neuroscientists identifying brain regions responsible for episodic memory and formulating a unified theory to explain it. The role of the amygdala also remained a source of confusion. Mishkin’s 1978 prominent study, concluded that damage to both hippocampus and amygdala structures was necessary for memory impairment as lesions restricted to either the amygdala or hippocampus alone did not cause significant memory loss [68]. These lesions also mirrored the suspected brain damage in the amnesic patient H.M. [6]. The study by Zola-Morgan, Squire, and their team corroborated Mishkin's earlier findings, confirming that extensive MTL lesions encompassing both the hippocampus and amygdala consistently resulted in severe memory impairments [69]. Anatomical evidence from the study by Insausti *et al.* identified that the entorhinal cortex (ERC) receives two-thirds of the cortical inputs from the perirhinal cortex (PRC), and parahippocampal cortex (PHC) [70]. This raised the possibility that PRC and PHC could have been responsible for episodic memory impairments, leading to a study identifying that lesions of PRC and PHC that spare the amygdala and hippocampal formation produce severe memory impairment [71]. All these studies led to the framework which is termed the MTL framework proposing a neural system comprising the hippocampus and closely interconnected regions, including the EHC, PRC, and PHC crucial for developing long-term memories [72].

The model suggested that activity in the neocortex reflects perception and short-term memory [73, 74]. However, to transform these memory traces into long-term storage, the MTL structures including the hippocampus, PRC, and PHC act as storage sites. These memory traces are then reconsolidated and reorganized in the cortical sites where they become less dependent on the hippocampus as discovered earlier in monkeys [75]. This model further suggests that the hippocampus has a limited capacity for storing memories, and the reorganization of memories to cortical sites happens to make the MTL system available for the acquisition of new memories [75, 76]. While the MTL model has enhanced our understanding of the contributions of MTL regions, it tends to isolate the MTL as a memory system separate from other brain systems. Indeed, connectivity studies have demonstrated that a distributed set of networks comprising cortical and subcortical brain regions contributes to episodic memory [77]. In addition, this framework mainly focuses on the acquisition of memories, while it provides little detail on how these memories are consolidated and later retrieved. It also does not provide details on the dynamic nature of episodic memories. Despite these limitations, the MTL framework remains a valuable starting point for understanding episodic memory.

### The PMAT Framework

"PMAT" framework proposes that MTL regions function as parts of two larger, interconnected networks: the posterior medial (PM) and anterior temporal (AT) systems [78]. According to the PMAT framework, the PM subnetwork comprises PHC, retrosplenial cortex (RSC), posterior cingulate (PCC), angular gyrus, precuneus, anterior thalamus, and mPFC, while the AT system includes regions such as the PRC, anterior ventral temporal cortex (aVTC), amygdala, and lateral orbitofrontal cortex (lateral OFC) [79–81]. The regions in the PM and AT subnetworks are connected to different regions of the ERC that provide an interface to communicate with the hippocampus [82]. For example, PHC showed preferential intrinsic functional connectivity with posterior medial ERC, while PRC with anterior-lateral ERC. The PM subnetwork engages in the online processing of contextual information and the long-term storage of previously learned contexts (for a detailed review, see [83]). On the other hand, the AT system is dedicated to processing item information and storing previously learned items in the form of concepts. These two systems interact together to support memory-guided behavior and two brain regions have been particularly implicated as the integration points in the PMAT framework. One is the hippocampus, which shows a strong connection with both systems, and the other is the ventromedial PFC, which is connected to regions in PM and AT, as well as the hippocampus [78].

**Fig. 3:**
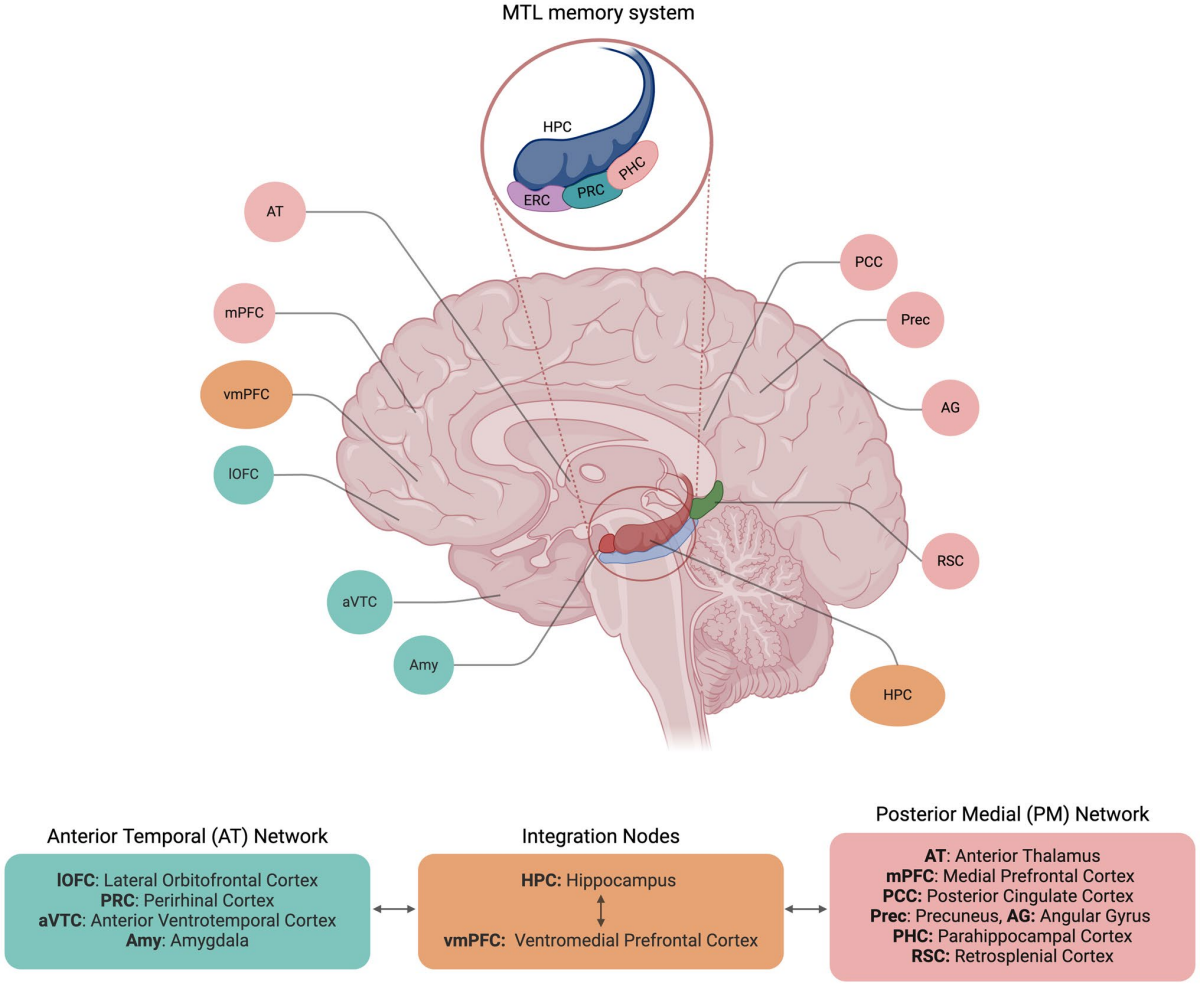
PMAT and MTL frameworks. The figure illustrates the regions identified in the PMAT framework. Green-highlighted boxes represent the AT network, while light maroon ones indicate the PM network. It also marks two integration nodes, the hippocampus and ventromedial prefrontal cortex, which communicate with each other and serve as an interface between the AT and PM networks. Additionally, the MTL memory system is highlighted, showing the regions included in this model. Created in BioRender. Khan, A. (2025) https://BioRender.com/b83o704.

The PMAT framework has proven to be instrumental in comprehending episodic memory processes and has served as a reliable guide in simulation studies [84, 85]. Importantly, the PMAT framework challenges the idea that the cortical regions either solely handle sensory processing or act as dedicated memory systems. Instead, they are multifaceted networks involved in a diverse range of cognitive functions including episodic memory. In essence, the PMAT framework highlights that memory is not confined to isolated brain regions within the MTL. It emerges from the interplay of these broader networks, each contributing to various cognitive aspects that underlie our ability to learn and remember. Fig. 3. identifies the brain regions involved in both PM and AT subnetworks while also outlines the MTL system. For a detailed review of the PMAT framework, see [86]

### The Default Mode Network and Beyond

The discovery of resting-state brain networks [87] was a significant event in the neuroscience field, which brought a substantial paradigm shift from traditional focal examinations to embracing a broader approach to investigating the dynamics of different brain networks. Particularly, the default mode network (DMN), a network of brain regions that is active during periods of rest and inactive during tasks, became a focal point of interest in various domains of cognitive neuroscience [87]. Comprising regions in the mPFC, MTL, as well as sections in the parietal cortex such as the inferior parietal lobule, PCC, and precuneus, DMN shows a significant overlap with the PMAT network. It is believed to mirror a mental time-traveling phenomenon when participants engage in thoughts about the past or future [88, 89]. DMN networks have been further deconstructed into specific subnetworks based on how they interact with distinct regions of the MTL. According to the connectivity studies, two parallel subnetworks exist: one involves the hippocampal network and is termed DMN-A, and the other involves the frontal network and is referred to as DMN-B [90, 91]. DMN-A is strongly connected to the posterior regions of the parietal cortex and is believed to play a role in subjective confidence during episodic recall, while DMN-B is connected to the anterior region of the parietal cortex and is engaged in semantic categorization [90]. Data-driven approaches have differentiated the Medial Temporal Network (MTN), consisting of MTL structures and precuneus, from the DMN [92, 93]. Recent work by Barnet *et al.*, suggests that the MTN facilitates communication between the visual network, DMN, and hippocampus [92]. These methods have further segregated the DMN into three subnetworks: the PM subnetwork, incorporating the PCC, RSC, angular gyrus, and DLPFC; the AT subnetwork, encompassing the temporopolar cortex, lateral OFC, and dorsal mPFC; and the medial prefrontal (MP) subnetwork, consisting of the mPFC and ERC. According to this framework, the PM and AT networks exhibit slightly distinct characteristics as described in the PMAT framework, notably the PHC and precuneus are not part of the PM subnetwork, and the PRC is not included in the AT subnetwork. In addition, the three DMN subnetworks exhibit varying connectivity along the hippocampal long axis and play distinct roles during episodic memory tasks. For example, MP is associated with emotion and value, connecting more to the anterior hippocampus, likely evaluating emotional valence and coordinating network activity. AT is involved in social cognition and theory of mind, processing social interactions and beliefs. Additionally, the PM subnetwork is related to control processes involved in episodic memory and self-referential processing. These networks work together depending on task demands.

Overall, these frameworks suggest that episodic memory networks can be approached through the lens of large-scale resting state networks.

## Brain Stimulation and Episodic Memory

**Fig. 4:**
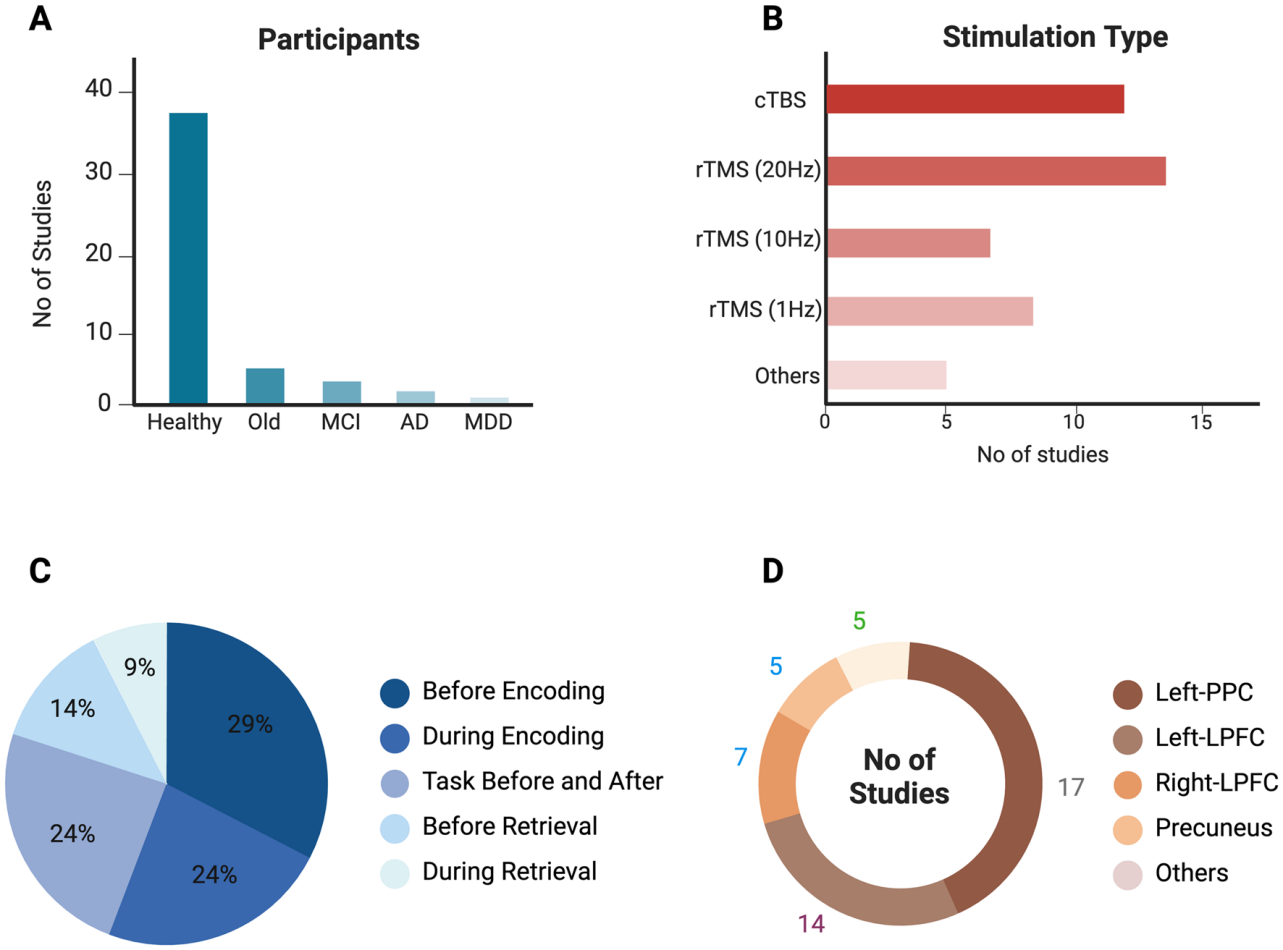
The summary of the included studies: **A** Depicts the demographics of participants in the included studies **B** Demonstrates the stimulation protocol employed in the included studies. **C** Shows the timing of when the brain region was targeted with respect to encoding and retrieval of memories, while some studies conducted the episodic memory task (encoding-retrieval) before and after stimulation referred to as task before and after. **D** Illustrates the percentage distribution of studies stimulating different brain regions involved in episodic memory processing. Different regions in the left posterior cortex have been combined together in the left-PPC. Created in BioRender. Khan, A. (2025) https://BioRender.com/c18m317.

Non-invasive brain stimulation presents an exciting possibility to comprehend and potentially enhance episodic memory function by targeting the regions and the network implicated in episodic memory. Deeper regions mentioned in the previous section, including the hippocampus, amygdala, and other MTL regions cannot be directly targeted using TMS. However, recent studies suggest that precise stimulation of cortical brain regions particularly in the frontal and parietal brain regions can indirectly modulate deeper brain structures by network-level communication. Thus, stimulation can have both local effects at the site of stimulation and network-wide effects throughout the cortico-subcortical network. We conducted a systematic review of studies utilizing TMS to modulate different regions of the episodic memory networks. A systematic search was conducted in two databases including PubMed and Web of Science. Further details about the selection of the studies are provided in supplementary materials and summary of included studies in provided in Fig. 4. In Table 1, we have briefly summarized the outcome of the studies utilizing TMS to modulate episode memory functions with the basic principles of TMS detailed below.

**Table 1:** The table summarizes the information extracted from the included studies including the target brain region, stimulation type, demographics of the participants, main behavioral and neuroimaging findings, and the timing of stimulation

Study	Target region	Stimulation type	Participants	Stimulation applied	Neuroimaging findings	Behavioral findings	
Blanchet *et al.* [128]	left or right DLPFC	Paired Pulse TMS	16 Healthy Young	During encoding	N	Right DLPFC stimulation enhances memory performance during full attention encoding and impairs it during divided attention encoding.	
Bonni *et al.* [118]	Precuneus, Left-PPC	cTBS	30 Healthy Young	Before Retrieval	N	Precuneus stimulation led to a decrease in source memory errors, while stimulation of the PPC had no such effect.	
Bonnici *et al.* [114]	Left-AG	cTBS	22 Healthy Young	Before Retrieval	N	Reduced free recall of autobiographical memories but had no effect on cued recall of autobiographical memories or recall of word-pair memories.	
Chen *et al.* [137]	Precuneus	rTMS (10Hz)	24 MCI	Memory tasks administered before and after stimulation	Modulation in hippocampal structure and increase in fMRI connectivity with the middle temporal gyrus.	Performance was restored after 4 weeks of rTMS treatment, with increased AVLT scores.	
Dave *et al.* [132]	Cerebellum	rTMS (5Hz) rTMS (20Hz)	25 Healthy Young	Before encoding	Beta stimulation led to increased N400 amplitude associated with semantic prediction, while theta stimulation modulated the ERP markers associated with encoding single words.	Theta stimulation improved episodic memory encoding, whereas beta stimulation did not influence episodic memory encoding.	
Dave *et al.* [116]	Left-PPC, Left-APC	cTBS	21 Healthy Young	Before encoding	The fMRI functional connectivity within frontal and hippocampal networks was differentially modulated by stimulating distinct parietal locations.	Left-PPC stimulation selectively reduced subjective recollection and resulted in subjects generating fewer details during tasks that require semantic simulation or divergent thinking.	
Davis *et al.* [120]	Left-DLPFC	rTMS (1Hz) rTMS (5Hz)	14 Healthy Young	Before encoding	The global connectivity and local connectivity were enhanced by 1 Hz and 5 Hz rTMS, respectively.	Memory performance was not significantly different during either rTMS condition when compared with baseline memory performance.	
Eick *et al.* (2020)	OFA	Triple TMS pulses	45 Healthy Young	During encoding	N	Stimulation reduced subsequent retrieval performance for the face-associated semantic information.	
Feurra *et al.* (2010)	Left-IFG and left-OFA	rTMS (10Hz)	12 Healthy Young	During encoding	N	Accuracy at retrieval showed a small decrease after left OFA stimulation, whereas stimulation over the left IFG reduced memory recall.	
Freedberg *et al.* (2021)	Left-IPC	rTMS (20Hz)	48 Healthy Young	Memory tasks administered before and after stimulation	Stimulation altered hippocampal-cortical connectivity, correlating with IPC-hippocampus pathway anisotropy, assessed via fMRI and DTI.	Stimulation resulted in a moderate effect size improvement in episodic memory, whereas procedural memory exhibited a small decrease.	
Freedberg *et al.* (2022)	Left-IPC	rTMS (20 Hz)	29 Healthy Young	Memory tasks administered before and after stimulation	N	Stimulation led to enhanced episodic memory performance on the day following the final stimulation session, but this effect did not persist beyond one week.	
Gagnon *et al.* [126]	left or right DLPFC	Paired pulse TMS	18 Healthy Young	During encoding or retrieval	N	Stimulation negatively impacted memory performance when applied to the left DLPFC during encoding and to the right DLPFC during retrieval, indicating lateralization effects in memory processing.	
Giglia *et al.* [135]	Right-DLPFC	rTMS (1Hz)	30 Healthy Young	Immediately after encoding or prior to retrieval	N	Stimulation of the right DLPFC disrupts late-stage encoding for long-term memory but has opposite effects during the pre-retrieval phase.	
Hawco *et al.* [122]	Left-DLPFC	rTMS (10 Hz)	40 Healthy Young	During encoding	N	High-strategy users exhibited decreased performance, whereas low-strategy users showed enhanced recall following stimulation.	
Hawco *et al.* [121]	Left-DLPFC	rTMS (10Hz)	17 Healthy Young	During encoding	The effect of stimulation at different onsets varied differently in distal brain regions with a temporal resolution in the hundreds of milliseconds as indicated by fMRI.		Associations for related pairs were better remembered than unrelated pairs.
Hebscher *et al.* (2019)	Precuneus	cTBS at 80% RMT for 40s 600Pulses, every 5Hz, 3 pulses at 30 Hz in each burst	23 Healthy Young	Before retrieval	Stimulation altered MTL-neocortical communication by modulating theta-gamma coupling measured by MEG.	Lower vividness ratings in memory tasks after stimulation	
Hebscher *et al.* [133]	Left-LPC	cTBS	20 Healthy Young	Before encoding	Stimulation resulted in an increased reinstatement of naturalistic stimuli fMRI activity patterns within the occipital cortex.	The accuracy of memory with a typical pattern was improved by hippocampal network targeted stimulation.	
Hermiller *et al.* [136]	Left-LPC	rTMS (20Hz)	16 Healthy Young	Memory task administered before and after 5 days of stimulation	N	Improved performance in face-word paired-associate recall memory test lasting 24 hours after stimulation	
Hermiller *et al.* [111]	Left-LPC	cTBS, iTBS, rTMS (20Hz), sham	24 Healthy Young	Before retrieval	Only cTBS affected the fMRI connectivity between the hippocampus and the memory-related HCN network.	cTBS significantly improved item retrieval success compared to sham and beta-frequency stimulation, whereas iTBS did not result in a significant improvement.	
Hermiller *et al.* [113]	Left-LPC	cTBS rTMS (20Hz)	16 Healthy Young	Trial-specific stimulation right before encoding	cTBS stimulation enhanced the fMRI activity of the specifically targeted left hippocampus during the encoding of scenes	cTBS leads to improved recollection.	
Hermiller *et al.* (2022)	Left-LPC	cTBS rTMS (20Hz)	15 healthy young 15 Old	Before encoding	After cTBS, young adults showed localized hippocampal network enhancement, whereas older adults experienced broader brain-wide connectivity increases.	cTBS improved memory in younger adults. In contrast, it did not benefit memory in older adults.	
Hill *et al.* [124]	Right-DLPFC	cTBS	36 (20 Healthy Young & 16 Old)	Before Retrieval	N	No significant intervention effect on task accuracy was observed in either of the age groups.	
Innocenti *et al.* [127]	left/right DLPFC	rTMS (10Hz)	18 Healthy Young	During encoding	N	Stimulation of Left-DLPFC disrupted memory performance while Right-DLPFC stimulation had no impact on memory performance.	
Kavanaugh *et al.* (2018)	Left DLPFC and bilateral DMPFC	rTMS (10Hz)	84 Treatment-Resistant Major Depressive Disorder	Memory tasks administered before and after stimulation	N	No stimulation effect on aspects of attention or working memory. The quality of episodic memory was better for the stimulation group.	
Koch *et al.* [119]	Precuneus	rTMS (20Hz)	14 early AD	Memory tasks administered before and after stimulation	An increased neural activity in beta band within the precuneus, and modulation in the functional connectivity between the PC and the medial frontal regions within the DMN.	Stimulation resulted in an enhancement of episodic memory, with no impact on other cognitive domains.	
Kahn *et al.*[79]	Left and Right VLPFC	Single Pulse TMS	14 Healthy Young	During encoding	N	left VLPFC stimulation impaired memory of familiar words, while right VLPFC stimulation facilitated subsequent memory of familiar words.	
Manenti *et al.* [134]	Left and right DLPFC	rTMS (20Hz)	31 Healthy Old	Before encoding or before retrieval	N	In the low-performance group, stimulation disrupted memory performance when left-DLPFC was stimulated before the encoding and not retrieval.	
Mangano *et al.* [117]	right/ left PPC	rTMS (1Hz)	40 Healthy Young	Before encoding or before retrieval	N	Stimulation on the right PPC before retrieval improved non-verbal recognition memory performance while stimulation before encoding did not influence memory performance.	
Nilakantan *et al.* [85]	Left-LPC	rTMS (20Hz)	16 Healthy Young	Memory task administered before and after 5 days of stimulation	EEG late-positive evoked potential amplitude and theta-alpha oscillatory power was reduced	Stimulation resulted in a lasting improvement in recollection precision for up to 24 hours.	
Sestieri *et al.* (2013)	Left-AG, Left-SPL	rTMS (20Hz)	16 Healthy Young	During retrieval	N	Memory performance was selectively disrupted following stimulation of the left AG relative to a sham condition or stimulation of the left SPL.	
Sole-padulles *et al.* [169]	Left-Prefrontal cortex	rTMS (5Hz)	40 Older adults with memory problems	Memory tasks administered before and after stimulation	Stimulation resulted in additional recruitment of right prefrontal and bilateral posterior cortical regions observed using fMRI	Stimulation resulted in an enhancement in associative memory performance	
Tambini and d'esposito, [40]	Posterior IPC	cTBS	22 Healthy Young	Before encoding	N	Stimulation enhanced associative memory success and confidence while Item memory was unaffected	
Tambini *et al.* (2018)	LOC	cTBS	58 Young Healthy	Immediately after encoding	Stimulation modulated hippocampal-lateral occipital cortex functional connectivity as assessed using fMRI	Stimulation selectively impaired associative memory retention.	
Thakral *et al.* [115]	Left-AG	rTMS (1Hz)	16 Healthy Young	Before encoding	N	Participants generated fewer episodic details for both future and past events and generated more non-episodic details for each class of event.	
Thakral *et al.* (2020)	Left-AG	cTBS	18 Healthy Young	Before encoding	A reduction in hippocampal activity during episodic simulation and divergent thinking	Stimulation reduced the number of episodic details produced for the simulation task and reduced idea production on divergent thinking.	
Turriziani *et al.*. [125]	Right-DLPFC	rTMS (1Hz) rtMS (50Hz)	100 Healthy Young and 8 MCI	Before retrieval	N	rTMS (1Hz) enhanced recognition memory in both Healthy and MCI participants. rTMS (50Hz) deteriorated memory performance.	
Van der plas *et al.* [123]	Left-DLPFC	rTMS (1Hz)	Healthy Young 40 (Experiment-1) 24 (Experiment-2)	During encoding	Stimulation decreased event-related power in the beta frequency band in posterior areas.	Stimulation enhanced memory performance indicated by more recalled words.	
Wais *et al. *(2018)	Left Mid-VLPFC Left-AG	rTMS (1Hz)	24 Healthy Elderly	Before encoding	N	Stimulation of mid-VLPFC diminished subsequent discrimination-based memory performance, no such effect was observed after stimulation of Left-AG.	
Wang *et al.* [15]	Left-LPC	rTMS (20Hz)	16 Healthy Young	Memory task administered before and after 5 days of stimulation	Stimulation increased fMRI connectivity of cortical-hippocampal network regions.	Stimulation improved associative memory performance up to 24 hours after the last stimulation session	
Warren *et al.* [84]	Left-LPC	rTMS (20Hz)	32 Healthy Young	Memory tasks administered before and after stimulation	Stimulation increased fMRI connectivity during the autobiographical retrieval phase.	Episodic memory improved after stimulation. This enhancement was predicted by task-specific increases in MTL connectivity.	
Yang *et al.* [170]	Left-AG	rTMS (20 Hz)	22 MCI & Alzheimer	Memory tasks administered before and after stimulation	In the aMCI group, stimulation increased fractional anisotropy in the right anterior thalamic radiation and refined thalamic functional network topology.	Stimulation improved episodic memory performance in MCI patients and AD patients.	
Ye *et al.* (2018)	Precuneus	rTMS (1Hz)	18 Healthy Young	Before encoding	N	Stimulation selectively impaired the metacognitive efficiency of memory judgment, but not perceptual discrimination.	

*DLPFC* dorsolateral prefrontal cortex, *PPC* posterior parietal cortex, *LPC* lateral parietal cortex, *VLPFC* ventrolateral prefrontal cortex, *IPC* inferior parietal cortex, *DMPFC* dorsomedial prefrontal cortex, *OFA* occipital face area, *APC* anterior parietal cortex, *LOC* lateral occipital cortex, *AG* angular gyrus, *RAVLT* rey auditory verbal learning test

### Basic Principle of TMS

**Fig. 5:**
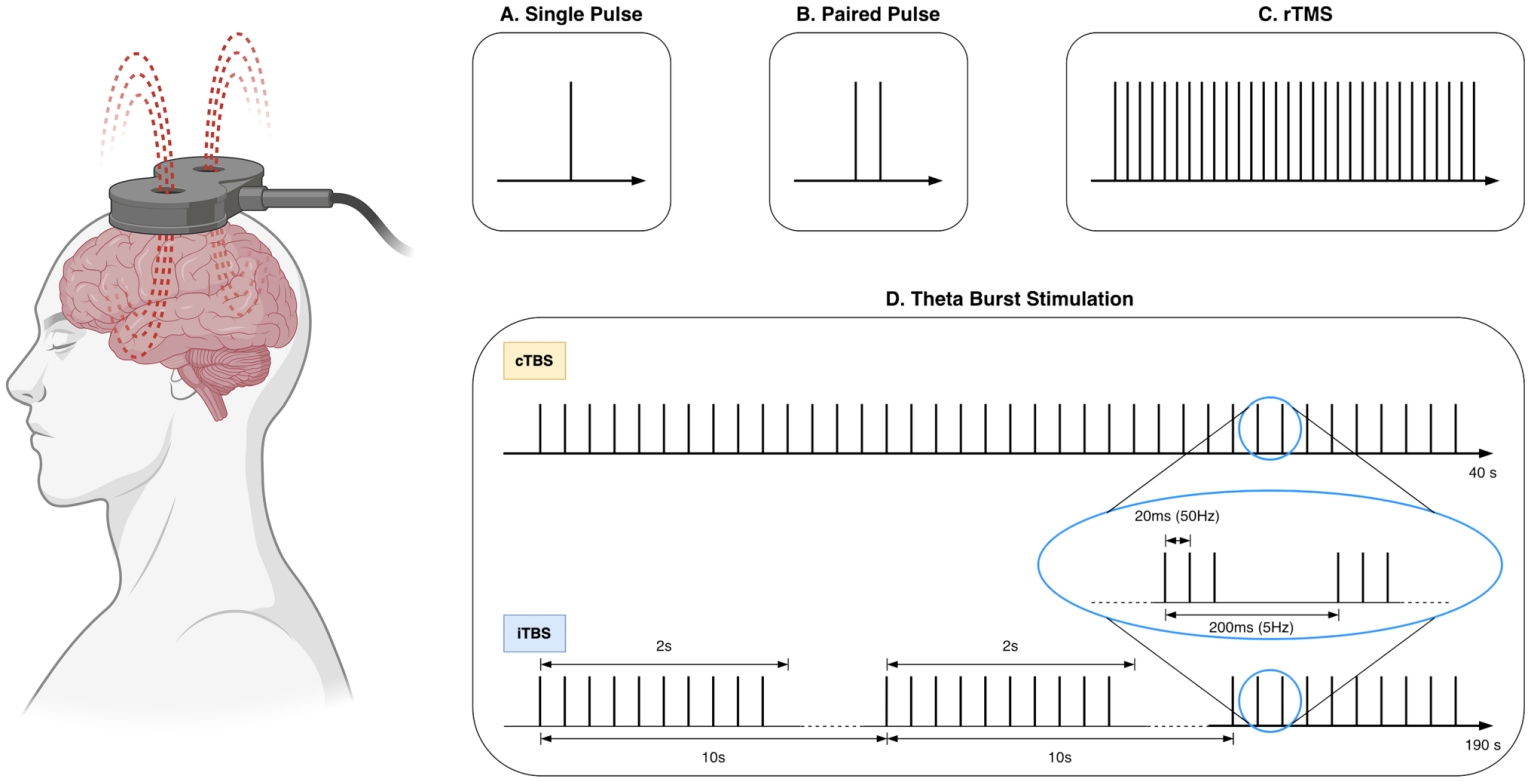
A summary of common TMS protocols: **A** Single pulse TMS. **B** Paired pulse TMS. **C** Repetitive TMS (rTMS). **D** Theta Burst Stimulation including cTBS and iTBS.

The scientific principle of TMS is based on the Faraday’s Law of electromagnetic induction. When a high-intensity electric current pulse passes through a magnetic coil, a magnetic field will be produced. The generated magnetic field penetrates the scalp and skull with minimal attenuation and induces an electric current pulse in the stimulated brain region as shown in Fig. 5. The intensity of this induced current can produce action potentials and activate brain networks. The induced current in the brain has an intensity proportional to the magnetic field and a direction opposite to the current in the coil. This induced electric current typically circulates up to a few centimeters from the external edge of the coil and flows in loops parallel to the coil plane. The intensity of the magnetic field decreases rapidly with increasing distance from the coil and is severely attenuated at deep brain structures such as the basal ganglia or thalamus. As a result, the TMS is mostly applied for stimulations of the cerebral cortex.

#### TMS Protocols

Different TMS protocols have been introduced since its inception. In single-pulse TMS, a single magnetic pulse is used to induce electrical currents in specific regions of the brain. One of the most common uses of single pulse TMS activation is the estimation of motor-evoked potential (MEP) elicited by stimulating the primary motor cortex [94] which is often used as a starting point for any experiment to determine the intensity of the stimulation. In a paired-pulse TMS, two TMS pulses are delivered at closely spaced intervals. This TMS protocol is usually applied to assess connectivity between and within cortical regions [95]. Repetitive TMS (rTMS) is a technique in which consecutive stimuli with a short interstimulus interval (ISI) can be applied [96]. The execution of rTMS usually needs a specific set of stimulators able to overcome the recharging time to maintain the same stimulus amplitude with incredibly short ISI. Conventionally, there are two main rTMS paradigms: low-frequency rTMS (LF-rTMS) and high-frequency rTMS (HF-rTMS). The LF-rTMS usually has a frequency of ≤ 1Hz and is believed to induce inhibitory effects due to long-term depression (LTD) of the synaptic transmission [97]. While the HF-rTMS, usually with a frequency of ≥ 5Hz, produces excitatory modulation to the targeted brain regions because of the long-term potentiation (LTP) [98–100]. Although the after-effect duration of rTMS can vary depending on the applied intensity, pulse number, and frequency, repetition of sessions can generally prolong the effect time [101]. Theta burst stimulation (TBS) is a patterned form of rTMS with more than one frequency pulse train. TBS usually comprises a burst of three 50 Hz pulses repeated with an ISI of 200 ms (5 Hz), a pattern resembling the natural theta rhythm in the hippocampus. TBS protocol can produce strong inhibitory or excitatory effects to modulate cortical activities. When stimulation is delivered in short bursts with pauses in between (for example 2 s of TBS followed by an 8 s pause), it is called an intermittent TBS (iTBS) protocol that can increase cortical excitability [102, 103]. However, if bursts are delivered continuously without pause, it is defined as a continuous TBS (cTBS), which produces an inhibition effect on targeted brain regions [104] (Fig. 5).

#### Neuro-Navigation of TMS

Introduced several years ago, navigation systems dedicated to TMS practice have served multiple purposes: (1) determining the precise cortical location of a TMS target, (2) guaranteeing the consistency of TMS targeting in repeated sessions or follow-up studies, (3) enhancing the precision of TMS motor mapping techniques; and (4) identifying the functional involvement of a cortical region (e.g., speech or motor ability), particularly in the context of presurgical mapping [105]. A more common technique to locate a brain region uses its spatial relationship with functional activities, like phosphenes or motor responses derived from the stimulation of the visual cortex or M1 respectively. In this method, the location of the TMS coil over the scalp will be adjusted continuously until the desired stimulation effect is observed; thus, the optimized coil location depends on the stimulation effects. Determining the exact brain regions to be stimulated requires an accurate online matching system between the stimulated site and the scalp coil location. One solution to this requirement is to adopt the international 10–20 EEG system [106]. By assuming there is a consistent correlation between scalp locations and underlying brain functional structures, the magnetic coil is placed over a specific 10–20 position to modulate neurons in the underlying cortex. Nevertheless, because this localization method ignores the inter- and intra-individual variation of cortical anatomy, comparing study results from different subjects can be challenging. Stereotaxic neuro-navigation devices offer a more precise solution to the coil placement problem by precisely positioning magnetic coils based on the underlying brain anatomy. Moreover, if the MRI guidance system uses individual MR images of a particular subject, it can fully account for the individualized brain structure. Combining MRI scanning with TMS, the frameless stereotaxic neuro-navigation system can guide the coil to a selected brain region on MR images [107]. The subject’s head and the MR scan can be co-registered in a common reference space using a set of anatomical landmarks that are visible both on the MRI and on the head (such as the tragus of the ear, the internal angles of both eyes and the alar wings of the nose). This offers a connection between MR images and real anatomy and a three-dimensional (3-D) orientation by interactive visual navigation. The landmark’s 3-D position can also be measured by a digitizing pen using a radiofrequency-based, mechanical, or optical tracking system. In an optical-tracking system, a camera measures the 3-D location of infra-red LEDs attached to the coil and the subject’s head, which enables simultaneous tracking of movement and orientation of the coil and the subject’s head in a 3-D space. Compared with non-navigated protocols, navigation methods increase the targeting accuracy from the order of centimeters to the order of millimeters [108]. Besides, the trial-to-trial coil replacement variability can be reduced to close to zero. These navigation systems are especially useful for targeting cortical regions other than motor-related cortices (for which the MEP recording offers a trustworthy targeting maker), such as the DLPFC [109]. Combined with navigation systems, robot arms can also be adapted to the TMS practice. In long-term treatment with multiple stimulation sessions, the robot arm navigated TMS can further increase the reliability and repeatability of the procedure [110].

### Insights from Stimulation Studies: Key Findings and Interpretations

Having established the involvement of several cortical and subcortical brain regions in episodic memory, within the MTL, PMAT, and default mode network frameworks, as well as understanding the fundamental principles of TMS-based brain stimulation, we now examine how stimulating specific cortical regions influences these networks. Additionally, we evaluate which framework offers a compelling explanation for the observed effects.

#### Stimulation of Regions in the Parietal Cortex

Numerous studies over the past twenty years have investigated the impact of stimulation on episodic memory nodes in an attempt to influence memory functions. However, it was not until 2014 that Wang *et al.* very precisely demonstrated that applying beta rTMS over a cortical region in the left-lateral parietal cortex (left-LPC), which exhibits robust connectivity with the hippocampus, modulates the connectivity not only with the hippocampus but also with regions within the PM network [15]. Surprisingly, moving the stimulation coil farther from the target cortical region diminished the effect of stimulation on fMRI connectivity. The same study reported an improvement in memory performance that lasted up to 24 hours after a 5-day stimulation paradigm. The findings were later replicated with a similar beta rTMS protocol in several other studies [84, 85, 111, 112]. In addition, the studies by Tambini *et al.* [112] and Hermiller *et al.* [113] utilized cTBS targeted at the same left-LPC location and demonstrated that even a single session can enhance episodic memory functions. Thus, both cTBS and rTMS (20 Hz), when applied to the left-LPC led to an improvement in episodic memory through modulation of the PM network. Interestingly, opposite effects have been found when stimulating the AG, a part of the PPC that has been implicated in various aspects of memory, including autobiographical memory and episodic detail generation. For example, [114] found that cTBS targeting the left AG selectively reduced the free recall of autobiographical memories. Furthermore, [115] demonstrated that rTMS to the left AG reduced episodic details for both past and future events. These results are consistent with the PMAT framework, where the AG is part of the PM network, involved in the retrieval and integration of contextual details. The findings also align with the network definitions based on the DMN, as discussed in the earlier section, since the regions targeted overlap within both frameworks.

In addition, Dave *et al.* investigated how anterior and posterior parietal cortex regions are distinctly involved in episodic memory processes [116] based on the two DMN subnetworks model proposed by Braga *et al.* [90]. Their study successfully demonstrated that stimulation differentially modulates the two memory subnetworks associated with different regions of the parietal cortex. A lateralization effect in the parietal cortex has also been reported. One particular study investigating the timing of stimulation and lateralization effects within the parietal cortex revealed that stimulating the right posterior parietal cortex (right-PPC) before retrieval practice, as opposed to the left-PPC, resulted in improved performance in non-verbal recognition memory tasks. Interestingly, the same study found that stimulating either the left or right PPC before encoding did not have any noticeable impact on memory performance [117]. This research lends support to the notion that the right PPC plays a distinct role in recognition memory. Within the parietal cortex, the precuneus is a crucial component of the DMN and is also part of the PM subnetwork according to the PMAT framework. A study by Bonni *et al.* used cTBS targeting the precuneus and observed a decrease in source memory errors, indicating improved context retrieval [118]. Additionally, another study by Koch *et al.* used beta rTMS on the precuneus and found that stimulation enhanced episodic memory [119].

**Fig. 6:**
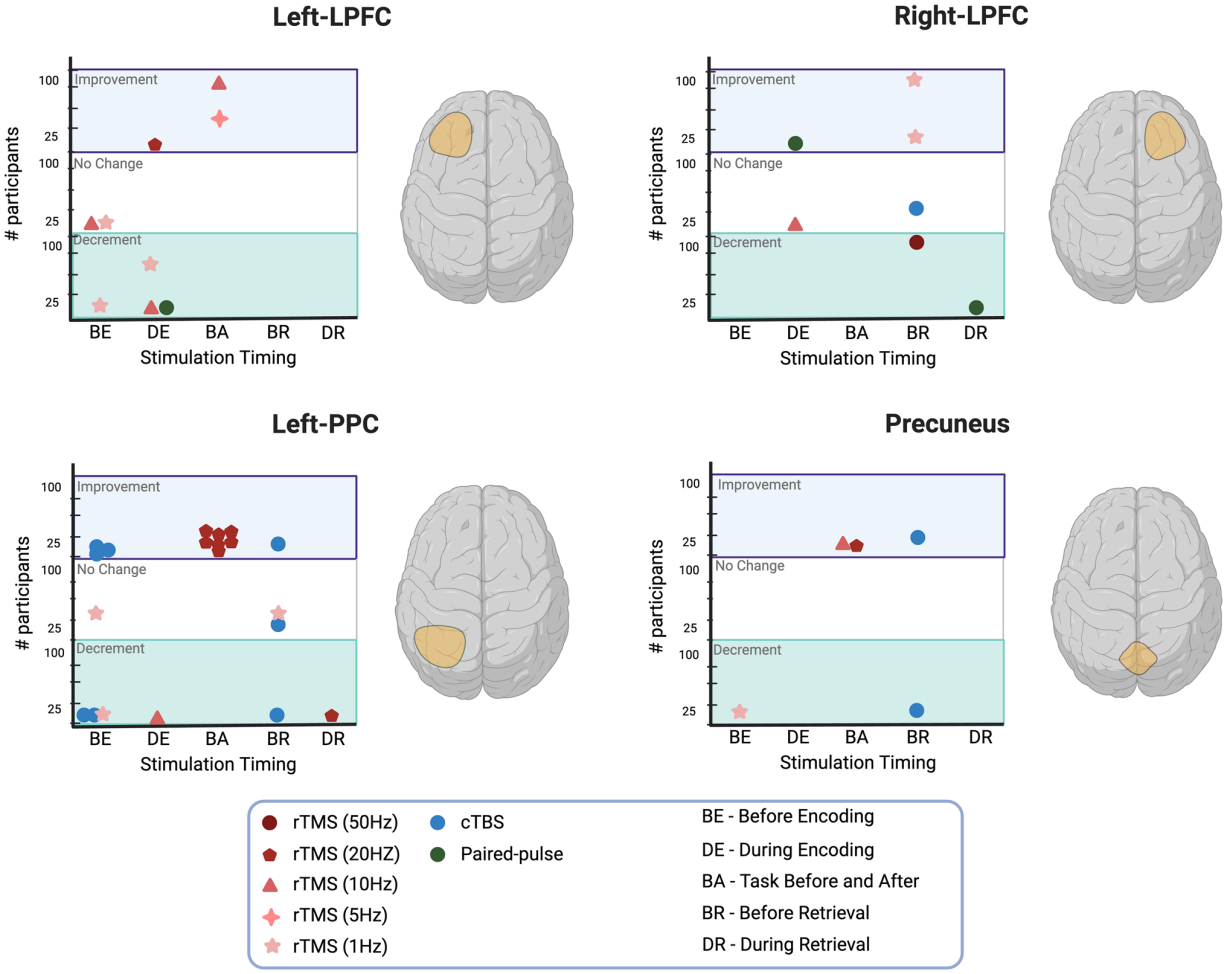
The figure summarizes the findings from the studies categorizing them into four primary brain regions: Left-LPFC, Right-LPFC, Left-PPC, and Precuneus. Each study is visually represented by a unique shape and color. For rTMS studies, specific shades of red are used in conjunction with distinct shapes. Non-rTMS studies are represented by non-red circles. The position of each circle on the graph reflects whether there was an enhancement, decline, or no observable change in behavior following stimulation. The y-axis illustrates the number of participants in each study, while the x-axis indicates the timing of when the brain region was targeted. Created in BioRender. Khan, A. (2025) https://BioRender.com/f81a360.

Thus, regions in the parietal cortex have shown promising results in causally manipulating episodic memory networks and potentially enhancing memory functions. The PMAT framework and DMN models provide valuable context for understanding these effects, suggesting that memory emerges from the interplay of multiple, distributed brain regions with both frameworks providing converging evidence on the role of the parietal cortex in episodic memory processes. The bottom panels of Fig 6. highlight studies that employ various stimulation protocols targeting regions in the parietal cortex including left-PPC and precuneus.

#### Stimulation of Regions in the Frontal Cortex

A good number of studies targeted regions in the frontal cortex including left and right DLPFC. While DLPFC is not considered a core part of the PMAT framework, it serves as a key node in the frontoparietal network (FPN), which is anti-correlated with the DMN. Particularly, the DLPFC regions have been implicated in executive control processes required for episodic memory encoding and retrieval. The earlier models suggested that left-DLPFC is involved in episodic memory encoding while right-DLPFC in episodic memory retrieval [20]. Thus, stimulation studies investigating encoding processes primarily targeted the left DLPFC [120–123], while those on retrieval focused on the right DLPFC [124, 125]. Some studies tested the lateralization effect by stimulating both regions during encoding and retrieval. For instance, Gagnon *et al.* investigated paired-pulse TMS applied to the left or right DLPFC during encoding and retrieval and found that memory performance declined when TMS was applied to the left DLPFC during encoding (compared to right-DLPFC and sham conditions) and to the right DLPFC during retrieval (compared to left DLPFC) [126]. Similarly, Innocenti *et al.* found that stimulating the left DLPFC before encoding impaired memory performance, whereas right DLPFC stimulation had no effect, supporting the lateralization model [127]. In contrast, Blanchet *et al.* reported that TMS applied to the right DLPFC enhanced memory performance during full attention encoding but impaired it during divided attention encoding [128]. The difference in the outcome could be attributed to the different stimulation protocols used in these studies. In addition, Davis *et al.* reported no significant difference in memory performance across rTMS conditions, including 1Hz and 50Hz, compared to baseline when left DLPFC was stimulated before encoding [120]. Among studies targeting right DLPFC before retrieval, Hill *et al.* found no significant effects with cTBS [124], whereas Turriziani observed memory improvement with 1Hz stimulation but disruption with 50Hz stimulation [125]. Overall, while some findings support a lateralization effect in prefrontal stimulation, others do not. The variability in stimulation protocols highlights the need for further research to clarify the role of prefrontal cortex regions in different stages of episodic memory processing. For a summary of findings from studies investigating prefrontal regions, particularly the left and right DLPFC using various protocols, refer to the top panels of Fig 6.

#### Stimulation of Other Brain Regions in Episodic Memory Network

Other regions like the occipital cortex and cerebellum have also been targeted in some studies, however, the supporting evidence for these interventions remains relatively weak. Notably, one important cortical brain region that has received little attention in stimulation studies on episodic memory is the mPFC which is a key node of the PM network according to DMN-based framework. Several investigations using electrical stimulation methods into the role of the mPFC in cognitive control have reported that stimulating this region can lead to alterations in inhibitory control during attention-related tasks [129, 130]. A recent study demonstrated that stimulation of mPFC potentially disrupts inhibitory control functions during memory retrieval resulting in retrieval of more non-target memories later on during the test phase [131]. However, these studies employed electrical stimulation methods, which come with certain limitations. Future research utilizing TMS could further investigate the possible involvement of the mPFC in processes related to episodic memory.

#### Timing-Dependent Effects of Brain Stimulation on Episodic Memory

While different brain regions can distinctly influence episodic memory functions, there are several other factors that contribute to the diverse effects of stimulation. We briefly discussed these factors in the previous section; here, we will expand on them further, with a particular focus on the role of stimulation timing. The influence of stimulation timing on memory performance can be observed in various studies as depicted in Fig. 6, which highlights how the application of stimulation before encoding, during encoding, before retrieval, during retrieval, and in studies where stimulation was nested between two blocks of episodic memory tasks, distinctly influences episodic memory processes.

Stimulation applied before the encoding phase altered memory encoding processes and the direction of the effect varied based on the stimulation protocol. For instance, cerebellar theta stimulation before encoding is reported to improve episodic memory encoding, whereas beta stimulation did not have the same effect [132]. Similarly, cTBS targeting the hippocampal network before encoding increased the reinstatement of naturalistic stimuli fMRI activity patterns within the occipital cortex, leading to improved memory accuracy for typical patterns [133]. In contrast, rTMS to the left DLPFC before encoding disrupted memory performance in low-performance groups, highlighting that the effect of stimulation can vary depending on individual baseline performance levels [134]. Applying stimulation during the encoding phase can also influence how information is processed and stored. For example, TMS applied to the right DLPFC during encoding is reported to enhance memory performance under full attention but impairs it under divided attention [128]. A study by Giglia *et al.* investigated how stimulation immediately after encoding influences episodic memory performance and reported that stimulation of the right DLPFC disrupts late-stage encoding for long-term memory [135]. Thus stimulation before, during, and immediately after encoding affects memory formation, as reflected in later retrieval. However, the direction of this effect varies based on stimulation frequency, and the underlying mechanisms require further investigation.

Stimulation applied before and during the retrieval phase modulated retrieval processes and accuracy. A study by Bonnici *et al.*, reported that cTBS to the left AG before retrieval selectively reduced free recall of autobiographical memories without affecting cued recall or word-pair memories [114]. Another study found that cTBS before retrieval improved item retrieval success compared to sham and beta-frequency stimulation, indicating that targeted stimulation can enhance retrieval accuracy [136]. Mangano *et al*. demonstrated that stimulation of the right PPC before retrieval improved non-verbal recognition memory performance, while stimulation before encoding did not have a significant effect, highlighting the importance of timing in targeting specific memory processes [117]. In contrast, Giglia *et al*. showed that rTMS with 1Hz of the right DLPFC during the pre-retrieval phase had opposite effects, enhancing retrieval performance [135].

In some studies, episodic memory tasks were conducted before and after stimulation. This protocol shares similarities with pre-encoding stimulation. However, because the stimulation effects may last until retrieval, it is challenging to determine if the observed impact is primarily due to effects on encoding, retrieval, or a combination of both. For example, Chen *et al.* observed that rTMS improved memory performance in MCI patients and modulated hippocampal connectivity [137]. Another study by Hermiller *et al.* noted that rTMS improved face-word paired-associate recall memory performance, lasting 24 hours after stimulation [111]. Similarly, Warren K *et al.* found that posterior-medial network-targeted stimulation increased fMRI connectivity during autobiographical retrieval phases, leading to enhanced episodic memory performance [84]. In summary, the timing of stimulation, whether applied before encoding, during encoding, before retrieval, during retrieval, or nested between memory tasks, plays a critical role in determining its impact on memory performance.

Another crucial aspect of episodic memory processing involves the temporal sequencing of various cognitive functions. For instance, during encoding, cortical brain regions are activated initially, guiding memory items toward MTL structures, and then MTL structures encode them. Similarly, during retrieval, control processes come into play during earlier time intervals to resolve interference, followed by the retrieval of memories occurring at a later stage after the onset of the event. In one study exploring this temporal dynamic in the brain, researchers applied TMS to the DLPFC at different points in the event processing stage while simultaneously collecting fMRI data [121]. This approach, combining TMS and fMRI, allowed for the investigation of dynamic brain connectivity with temporal precision. Results revealed distinct timing effects in brain regions linked to various cognitive processes, highlighting the potential of TMS-fMRI for capturing rapid connectivity dynamics.

The prominent behavioral findings from the studies have been visually demonstrated in Fig. 6, categorizing stimulation impact into four target brain regions including, left-LPFC, right-LPFC, left-PPC, and precuneus. The studies collectively highlight that the choice of stimulation target, stimulation type, and the timing of stimulation can differentially influence memory performance and neuroimaging outcomes. The varying effects observed in these studies emphasize the complexity of episodic memory and suggest that the optimal approach may depend on individual factors and experimental context. Despite the variability in findings across studies, one consistent effect emerged: stimulation of the Left-PPC improved memory performance when administered before a memory task.

## Challenges and Future Directions

TMS holds promise for investigating and modulating the neural networks of episodic memory, but several challenges require careful consideration. One major challenge is the variability in the outcome of TMS. It can sometimes produce contrasting effects even when targeting the same brain region with similar stimulation protocols (see Fig. 6). Factors contributing to this variability include, but are not limited to, individual differences in brain structural and functional organization, brain state, motor threshold determination, and others [138–140]. In this section, we discuss these factors in detail and provide guidelines for future studies that could pave the way for reproducible TMS effects.

### Individual Differences in Structure and Function

Individual differences in brain structural and functional organizations influence how stimulation affects the targeted brain regions, leading to inconsistent effects across individuals [139, 141]. A network-based approach, as it has been used in several studies included in our review provides a solution to individualize brain stimulation to enhance its efficacy. However, most of the existing methods do not account for variations in brain networks that happen over time and between resting and task states, and they are largely based on resting state networks. Another factor influencing the effect of stimulation on a specific brain region is its interaction with the ongoing neural oscillatory activity of that region or the brain state [140]. Functional brain states fluctuate both across individuals and within individuals over time [142–144], which can significantly influence the effects of TMS. Precision fMRI, which involves extensive data collection from single individuals, offers a possible solution [145]. By providing more detailed and personalized data, that incorporates changes in both resting and task brain state, the effectiveness of techniques like TMS can potentially improve in both understanding and enhancing memory capabilities.

In addition to using precision fMRI to understand these brain fluctuations, other imaging methods can be utilized. For example, the brain states may reflect in the oscillatory activity that can be observed at the scalp electrodes using EEG/MEG, and these oscillations, particularly in theta, alpha, beta, and gamma frequency bands are linked to various aspects of cognition [146, 147]. Each frequency band, depending on the region, contributes uniquely to specific cognitive tasks [148]. For instance, theta activity in the frontal cortex is thought to contribute to control processes reflecting the interplay between hippocampal and prefrontal brain regions required during encoding or retrieval, while beta and gamma band activities in the same region reflect top-down and bottom-up attentional mechanisms, respectively [2]. Furthermore, alpha and beta desynchronization in the parietal brain regions is thought to reflect successful encoding and retrieval of memories [149]. TMS can exploit different frequencies that can entrain the ongoing oscillatory frequency bands associated with different aspects of cognition.

Interestingly, these oscillatory activities interact with each other to give rise to complex cognitive processes. For example, theta-gamma coupling in the hippocampus is thought to contribute to episodic memory processes. Specifically, the phase of theta-gamma coupling during encoding and retrieval is indicative of whether a memory is successfully remembered or forgotten [150]. Thus, a promising avenue might involve using theta-coupled gamma stimulation to modulate hippocampal function, as this specific coupling is thought to be crucial for episodic memory. In addition, converging evidence has supported the notion that slow wave activity including theta and delta bands during sleep is responsible for the consolidation of memories [151]. Therefore, in the context of brain stimulation studies, understanding the impact of stimulating a specific brain region with a particular frequency relies on identifying the inherent oscillations of that region. Ideally, future studies should first identify the natural oscillatory patterns of the target region before implementing stimulation. As this inherent oscillation fluctuates over time, ongoing adjustments to the stimulation protocol are necessary to achieve the most personalized and effective treatment. In line with this, Ezzyat *et al.* reported that closed-loop stimulation of the lateral temporal cortex, guided by real-time brain state decoding to adjust frequencies, can improve memory encoding and recall [152].

In addition, the idea that stimulation frequencies can be excitatory or inhibitory initially emerged from studies on motor function. However, that does not seem to be true in the case of episodic memory functions. For example, the study by Turriziani *et al.* employed 1Hz rTMS, targeting the right DLPFC, and reported enhanced cognitive performance in recognition memory [125]. Hence, the effects of TMS can vary depending on the specific brain region being stimulated and the categorization of TMS frequencies as excitatory or inhibitory should be made with caution, as their effects are dependent on the stimulated brain region and brain function being studied. (For a detailed systematic review of the effects of excitatory and inhibitory non-invasive brain stimulation on PFC please see [153]).

### Motor Threshold and Cortical Excitability

In addition, while the method for determining motor threshold is somewhat standardized [154, 155], it still requires manual adjustments, which can affect how stimulation interacts with the cortex and lead to varying effects across participants. More importantly, this threshold is determined based on motor-evoked potential and then used to stimulate other non-motor-related brain regions while assuming that cortical excitability remains consistent across different brain areas. However, this is not the case, cortical excitability varies across brain regions and changes over time [156]. Therefore, directly measuring brain activity at the target site is crucial for determining the appropriate stimulation intensity. Integrating TMS with EEG and other neuroimaging techniques offers a more reliable approach to assessing cortical excitability [157]. TMS intensity-dependent effects on EEG signals have been reported to provide accurate measures of regional cortical excitability, particularly for non-motor brain areas [158].

### Dynamic Perspective on Episodic Memory Networks

Overall, current frameworks, while valuable, treat episodic memory networks as somewhat static. Because episodic memory unfolds across space and time, a more dynamic understanding is needed. Techniques like dynamic functional connectivity that index changes in neural activity patterns over time can map these evolving interactions [159]. Data-driven approaches can also refine the understanding of these networks [160]. In addition, episodic memory involves multiple stages (encoding, short-term maintenance, consolidation, and retrieval), but current research primarily focuses on encoding and retrieval, leaving short-term maintenance and, critically, consolidation relatively unexplored. Future studies could bridge this gap by examining the causal effects of TMS on episodic memory networks by delivering stimulation during the short-term maintenance period or even during the pre-sleep, wakeful rest period, or during sleep, which is known to be critical for memory consolidation.

### Other Forms of Brain Stimulation

While our study and other reviews have highlighted the distributed nature of episodic memory, involving numerous brain regions, most of the current stimulation techniques, including TMS, can only indirectly target non-cortical areas. This technical limitation hinders the ability to directly study deeper brain regions that play a critical role in episodic memory processing. Recent advancements in non-invasive deep brain stimulation have opened a new venue of research for targeting deeper structures directly. For example, temporal interference stimulation (TIS), a form of electrical stimulation, has been introduced which involves the simultaneous application of two high-intensity and high-frequency electrical currents through the scalp [161]. These currents are strategically administered in a manner that results in their interference within a deeper brain region, generating a new current with a frequency equivalent to the difference between the two originally applied currents. What makes this form of stimulation intriguing is that, at these high frequencies, neurons remain unstimulated, and the impact within the brain occurs only at the point where the two currents intersect. This characteristic offers an excellent means of maintaining control for placebo effects. A recent study utilizing TIS stimulation targeted at the human hippocampus successfully demonstrated that distinct regions of the hippocampus can be focally modulated using TIS by steering the current ratio of the pair of electrodes [162]. The study also reported an enhancement in memory function in addition to modulation of functional connectivity between the AT network and the hippocampus. Building on a similar idea, magnetic-TIS has also been recently introduced in which high-frequency magnetic fields are used instead of electric fields [163]. However, these techniques are still in their infancy stage and future studies could explore their potential.

This review did not include studies utilizing different forms of TES. However, several other systematic reviews have demonstrated that such current-based stimulation methods can potentially affect a range of cognitive functions [164, 165]. A recent meta-analysis examining the impact of anodal (positive polarity stimulation) and cathodal (negative polarity stimulation) transcranial direct current stimulation (TDCS) on episodic memory found the effects to be small and non-significant [166]. In contrast, another meta-analysis focusing on episodic memory in older adults reported that anodal stimulation led to significant memory improvements compared to sham stimulation, both immediately after stimulation and at long-term follow-up [167]. One limitation of conventional TES techniques is their lack of focal precision, as their effects tend to be dispersed. Research has shown that the maximum achievable field focality is constrained by head anatomy and its physical properties, highlighting a trade-off between focality and intensity [168]. As a result, they may not be ideal for investigating the functions of highly localized brain regions or networks. Nevertheless, they remain valuable for clinical applications due to their user-friendly nature.

### Clinical Applications and Future Prospects

Finally, the clinical impact of TMS is of particular importance, especially considering the increasing prevalence of memory impairments and the need for effective, non-invasive treatment options. Although the majority of the studies we identified were conducted on healthy young participants, there are some studies showing that stimulation can result in an enhancement in episodic memory in the elderly [169] and in patients with memory impairments [119, 170]. One notable finding across studies on healthy participants was that stimulating regions in the left PPC prior to a memory task leads to an improvement in memory performance. While the mechanisms underlying these enhancements remain inadequately understood, they show the potential of using TMS as a clinical instrument for memory impairments. Further research in this area could lead to new interventions for improving memory function in populations with memory deficits, such as patients with Alzheimer's disease or other forms of dementia.

Future brain stimulation studies should adopt a multifaceted approach to explore how episodic memory networks interact and how their connectivity evolves during various episodic memory processes, incorporating a personalized perspective to account for individual differences. Additionally, integrating resting-state networks with task-based fMRI and other neuroimaging techniques (MEG, EEG) is crucial for capturing real-time brain function and gaining deeper insights into how these networks operate during active memory tasks. Closed-loop brain stimulation methods that monitor brain activity and adapt stimulation in real time offer a promising approach to understanding these dynamics.

## Supplementary Information

Below is the link to the electronic supplementary material.

Supplementary file1 (PDF 260 kb)
