# Supplementary material for: From Correlation to Causation: Understanding Episodic Memory Networks
Source: Neurosci Bull. 2025 Jun 24;41(8):1463–86. doi: 10.1007/s12264-025-01407-2 (PMC12314160; doi:10.1007/s12264-025-01407-2)

Protocol Registration

In accordance with PRISMA guidelines, the protocol was registered with the Prospective Register of Systematic Review (PROSPERO), under the identification number CRD42023404332.

Literature Search

We independently conducted searches on two databases, namely PubMed and Web of Science, to identify studies that met the inclusion criteria. The search was performed using the following keywords.

SC1 (Transcranial Magnetic Stimulation OR TMS) AND (episodic memory OR long-term memory OR episodic-memory OR long-term memory) AND (network)

Inclusion Criteria

The analysis included studies that met the following criteria: (1) Randomized controlled trials
SC2 utilizing transcranial magnetic stimulation (TMS) for a single session or multiple sessions; (2) Parallel, longitudinal or crossover study design; (3) Single/double/triple blinded studies comparing tMS stimulation to a control group receiving sham stimulation. If the intervention involved another component, such as cognitive training, the comparison was made between intervention plus cognitive training and sham plus cognitive training; (4) Published in an international peer-reviewed journal until February 28, 2023; (5) Published in the English language.

Main Outcomes

The systematic review focused on identifying studies that utilized fMRI to demonstrate modulation of episodic memory networks and behavioural results, and studies that used fMRI for identification of episodic memory networks and reported only behavioural results.

Data Extraction

Following the identification of studies through the search strategy, duplicate studies were removed using EndNote 20. After the removal of duplicates, the titles of the remaining studies were evaluated, eliminating those not meeting the inclusion criteria. The second round of evaluation was performed on the abstracts and studies not meeting the inclusion criteria were removed. After screening of titles and abstracts, full articles were assessed. After the selection of studies, the data including study design, sample size, participants characteristics, type of TMS intervention, and behavioural and neuroimaging outcomes were extracted.

Search Results

Created in BioRender. Khan, A. (2025) https://BioRender.com/f30j839

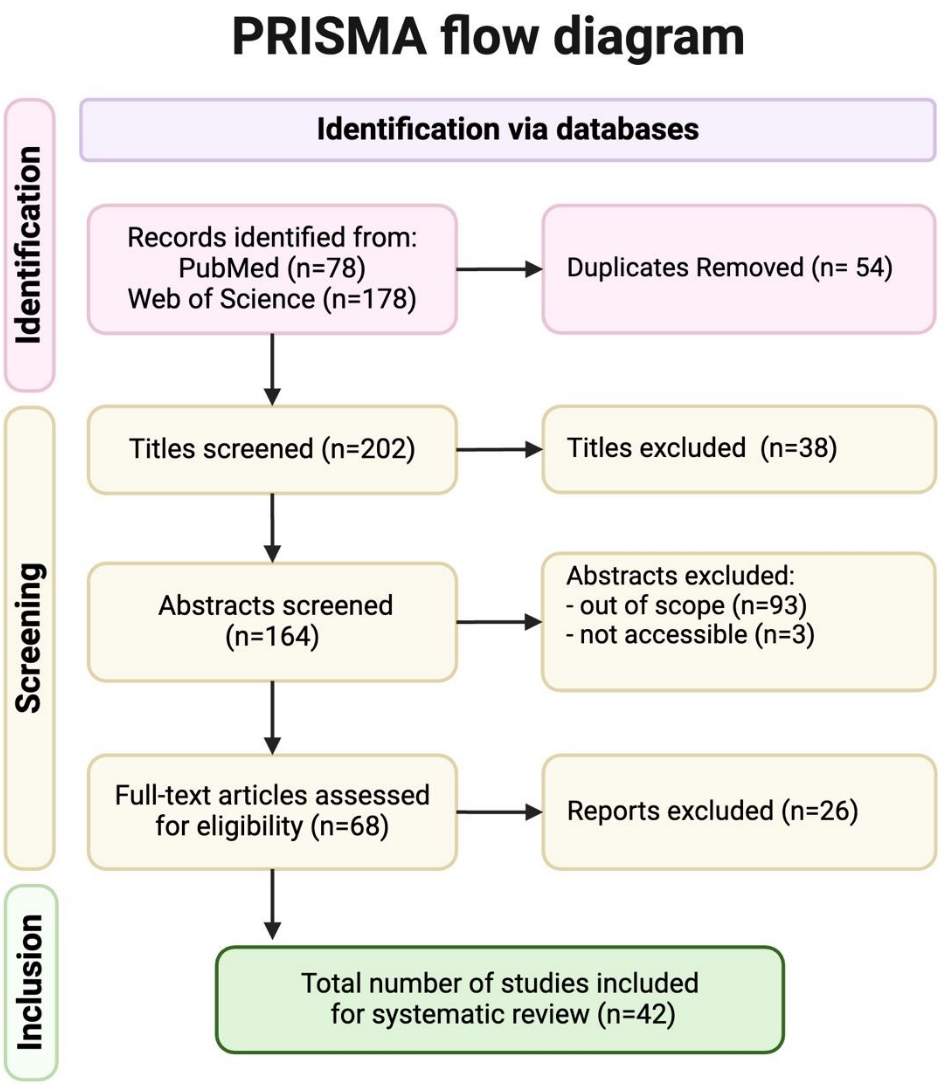

Supplement: Supplementary file 1 — Supplementary file1 (PDF 260 kb) [file 12264_2025_1407_MOESM1_ESM.pdf]
